# Importance of diagnosis-to-treatment interval in newly diagnosed patients with diffuse large B-cell lymphoma

**DOI:** 10.1038/s41598-021-82615-4

**Published:** 2021-02-02

**Authors:** Masahiro Yoshida, Yosuke Nakaya, Katsujun Shimizu, Naoko Tatsumi, Minako Tsutsumi, Hoyuri Fuseya, Mirei Horiuchi, Takuro Yoshimura, Yoshiki Hayashi, Takafumi Nakao, Takeshi Inoue, Takahisa Yamane

**Affiliations:** 1 Department of Hematology, Osaka City General Hospital, Osaka, Japan; 2 Department of Pathology, Osaka City General Hospital, Osaka, Japan

**Keywords:** Oncology, Cancer

## Abstract

Treatment of patients with malignancy sometimes be delayed due to various reasons. Several studies revealed that an influence of diagnosis-to-treatment interval (DTI) on outcomes differs depending on the type of malignancy. In this study, we evaluated the influence of DTI on clinical outcomes in newly diagnosed patients with diffuse large B-cell lymphoma (DLBCL). A total of 199 patients were identified with a median DTI of 22 days. At 2 years, patients with short DTI (0–22 days) showed significantly poorer OS (62.7% vs 86.4%) and PFS (55.1% vs 75.9%) compared to those with long DTI (over 22 days). Although short DTI was strongly correlated with several known adverse factors, it remained to be an independent prognostic factor by multivariate analysis. In conclusion, our study confirmed the importance of DTI in patients with DLBCL. Researchers should consider DTI as one of the important prognostic factors and plan clinical trials to be able to enroll patients with aggressive disease requiring urgent treatment.

## Introduction

Diffuse large B-cell lymphoma (DLBCL) is the most common subtype of non-Hodgkin’s lymphoma. It typically presents with aggressive behaviour, progressing over months, and becomes fatal without treatment^1^. It can be cured with immunochemotherapy by rituximab, cyclophosphamide, doxorubicin, vincristine, and prednisolone (R-CHOP)^2^. Although the International Prognostic Index (IPI), which incorporates clinical variables such as age, Ann Arbor stage, extranodal lesions, high serum lactate dehydrogenase (LDH), and performance status (PS), is a valuable prognostic tool, newer prognostic factors continue to be reported^1^.

Treatment of patients with malignancy sometimes can be delayed due to various reasons (e.g., additional examination, enrolment in clinical trial, patient’s social problem). Several studies revealed that an influence of diagnosis-to-treatment interval (DTI) on outcomes differs depending on the type of malignancy^3^. For breast cancer^4,5^ and colorectal cancer^4,6^, long DTI is reported to be associated with poor overall survival (OS). On the other hand, short DTI is associated with poor OS for lung cancer^7^.

Several studies reported the association between short DTI and poor outcomes in patients with DLBCL^8–12^. However, other studies reported opposite result^13^ or no association^14^, and the influence of DTI to outcomes in patients with DLBCL has been controversial. In the present study, we evaluated the influence of DTI and other parameters on clinical outcomes in newly diagnosed patients with DLBCL.

## Methods

### Patients

We conducted a retrospective analysis using a database from our hospital. Patients were eligible if they are aged 20 years or older, are newly diagnosed with DLBCL between April 2007 and March 2017, and have received CHOP or dose-reduced CHOP-like regimen with or without rituximab. Patients with HIV infection, central nervous system lesion, intravascular lymphoma, primary mediastinal large B-cell lymphoma, and transformation of indolent lymphoma, who did not receive anthracycline-containing chemotherapy, and who received treatment before pathological diagnosis were excluded from analysis. The timing and choice of treatment were left to the physicians’ clinical judgment. This study was approved by the Institutional Review Board of the Osaka City General Hospital.

### Treatment

At our hospital, we usually employ a CHOP-based regimen (cyclophosphamide 750 mg/m^2^, doxorubicin 50 mg/m^2^, and vincristine 1.4 mg/m^2^ on day 1 and prednisolone 100 mg/day on days 1–5) for patients younger than 70 years and a dose-reduced THP-COP-based regimen^15,16^ (cyclophosphamide 500 mg/m^2^, pirarubicin 30 mg/m^2^, and vincristine 1 mg/m^2^ on day 1 and prednisolone 30 mg/day on days 1–5) for patients over this age. The dose-reduced THP-COP regimen for the elderly was revealed to have a similar effect to that of a CHOP regimen for younger patients in our previous study^15^.

### Statistical analysis

The endpoints of this study were OS and progression-free survival (PFS). The OS was defined as the time from diagnosis to the last visit with the patient or death from any cause. The PFS was defined as the time from diagnosis to disease progression or death. Data were analyzed as of June 2018. DTI was defined as the time from report date of pathological diagnosis to the initiation of treatment. Comparisons of baseline characteristics were performed using Fisher’s exact test for categorical variables and Student’s *t*-test for continuous variables. The Kaplan–Meier method was used to evaluate OS and PFS, and the log-rank test was used to compare the differences. Univariate analysis was performed using a Cox proportional hazards model. Factors independently associated with OS or PFS were identified by multivariate analysis using a Cox proportional hazards model in a stepwise fashion. Prior to using multivariate analysis, we integrated factors to stabilize the analysis and to decrease multicollinearity among highly correlated variables that included age, Ann Arbor stage, extranodal lesions, high serum LDH, and PS into the IPI score. The hazard ratios (HRs) and corresponding 95% confidence intervals (CI) were also obtained. p-values < 0.05 were considered statistically significant. The above statistical analyses were performed with EZR^17^ (Saitama Medical Center, Jichi Medical University, http://www.jichi.ac.jp/saitama-sct/SaitamaHP.files/stat-medEN.html), a graphical user interface for R (version 3.1.0; The R Foundation for Statistical Computing, Vienna, Austria). More precisely, it is a modified version of R commander (version 2.0-4), designed to add statistical functions frequently used in biostatistics. The evaluation of nonlinear association with OS and DTI was performed with Microsoft Excel 2016 (version 16.16.19; Microsoft Corporation, Redmond, WA, USA).

### Ethics approval

This study was performed in line with the principles of the Declaration of Helsinki. Approval was granted by the medical ethics committee of the Osaka City General Hospital.

### Consent to participate

Informed consent was obtained by the opt-out method on the web-site.

### Consent for publication

Informed consent was obtained by the opt-out method on the web-site.

## Results

### Patient characteristics

A total of 199 patients were identified (Table 1), with a median age of 70 years (range 28–87). 81% were > 60 years, 51% were male, 68% were Stage III/IV, 60% had IPI score 3–5, and 16% were diagnosed in other facilities. Forty-eight percent were treated with CHOP, 52% were given dose-reduced THP-COP regimen, and 94% were combined with rituximab. The reasons for omitting Rituximab were CD20 negativity (n = 1), severe infusion reaction (n = 1), early death before using Rituximab (n = 2), concerns about tumor lysis syndrome (n = 4), and details unknown (n = 4).

**Table 1:** Patient clinical and laboratory characteristics.

	Short DTI (0-22 days)	Long DTI (over 22 days)	All patients	p
	(*n* = 102)	(*n* = 97)	(*n* = 199)	
Men/women	51/51	50/47	101/98	NS
Age (mean)	70	66	70	0.01
PS 2–4	46 (45%)	12 (12%)	58 (29%)	< 0.001
B symptoms	33 (32%)	15 (15%)	48 (24%)	0.007
Serum albumin (mean, g/dl)	3.2	3.6	3.4	< 0.001
LDH > ULN	86 (84%)	64 (66%)	150 (75%)	0.003
Ann Arbor stage III–IV	79 (77%)	56 (58%)	135 (68%)	0.004
Extranodal lesions	71 (70%)	60 (62%)	131 (66%)	NS
Bulky lesion	19 (19%)	16 (16%)	35 (18%)	NS
IPI 3–5	74 (73%)	45 (46%)	119 (60%)	< 0.001
Diagnosed in other facilities	12 (12%)	19 (20%)	31 (16%)	NS
**Treatment**				
CHOP based	44 (43%)	52 (54%)	96 (48%)	NS
THP-COP based	58 (57%)	45 (46%)	103 (52%)	NS
Rituximab	95 (93%)	92 (95%)	187 (94%)	NS

*PS* performance status, *LDH* lactate dehydrogenase, *ULN* upper limit of normal, *IPI* International Prognostic Index.

**Figure 1:**
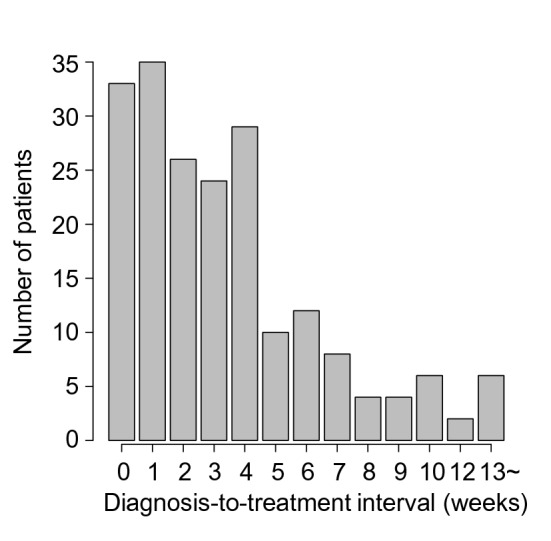
Distributions of diagnosis-to-treatment interval.

A median DTI was 22 days (range 0–393) (Fig. 1). Seventy-five percent of patients received treatment within 36 days from pathological diagnosis and 97% within 100 days. Six patients received treatment 100 days after diagnosis. Although the cause of treatment delay was not fully recorded, the reason of the delay for these six patients seems to due to comorbidities (n = 5) and poor economy (n = 1). When we divided patients into two groups by DTI, short DTI (0–22 days) was associated with clinical factors such as older age, poorer PS, B symptoms, lower serum albumin, elevated LDH, higher Ann Arbor stage, and higher IPI. Sex, extranodal lesions, bulky lesion, facility of initial diagnosis, and treatment regimen were not associated with DTI.

### Overall and progression-free survival

With a median follow-up of 1091 days (range 31–3951), 69 patients had died and 88 patients had PFS events (75 progression and 13 death without progression). The 2-year OS and PFS were 74.6% and 65.4%, respectively. The median survival was not reached. The median PFS was 91 months.

**Figure 2:**
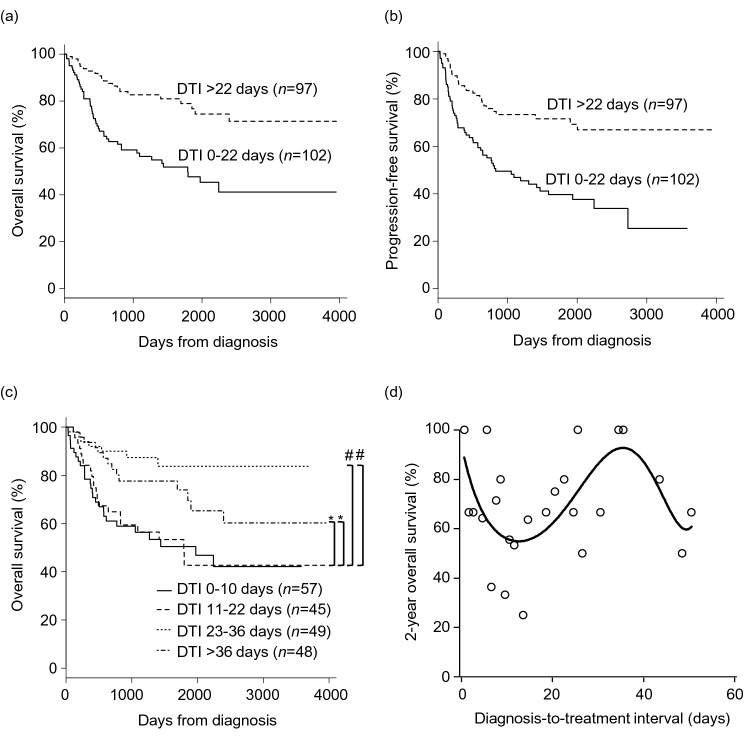
Survival of patients. Kaplan–Meier curves of overall survival (OS) (**a**) and progression-free survival (**b**) by diagnosis-to-treatment interval (DTI) divided into two groups. (**c**) Kaplan–Meier curves of OS by DTI divided into quartile. *p < 0.001; ^#^p < 0.05. (**d**) Two-year OS according to DTI. Nonlinear relationship was modeled using polynomial regression.

At 2 years, patients with short DTI (0–22 days) showed significantly poorer OS (62.7% vs 86.4%; p < 0.001, Fig. 2a) and PFS (55.1% vs 75.9%; p < 0.001, Fig. 2b) compared to those with long DTI (over 22 days). When we divided patients into quartile by DTI, shorter DTI (0–10 days,11–22 days) remained to be associated with significantly poorer OS compared to longer DTI (23–36 days, over 36 days, Fig. 2c). Patients with DTI of 23–36 days tend to show better OS compared to those with DTI of over 36 days (p = 0.108). We observed a nonlinear association with 2-year OS and DTI (Fig. 2d). The 2-year OS was lowest for patients with DTI of 5–15 days, highest for those with DTI of 30–40 days, and decreased afterward.

### Univariate and multivariate analysis

Univariate analysis showed that the parameters negatively affecting OS and PFS were as follows: short DTI (0–22 days), poorer PS, B symptoms, lower serum albumin, elevated LDH, higher Ann Arbor stage, extranodal lesions, higher IPI score, and nonuse of rituximab (Table 2). Older age and dose-reduced THP-COP regimen negatively affected OS alone.

**Table 2:** Factors associated with survival by univariate analysis.

Factor	Overall survival		Progression-free survival	
	Hazard ratio (95% CI)	*p*	Hazard ratio (95% CI)	*p*
Sex (male)	1.16 (0.72–1.87)	0.53	1.006 (0.66–1.53)	0.98
Age	1.03 (1.006–1.056)	0.02	1.019 (0.998–1.041)	0.07
PS 2–4	2.69 (1.66–4.35)	< 0.001	2.42 (1.57–3.72)	< 0.001
B symptoms	1.71 (1.022–2.86)	0.04	1.55 (0.98–2.45)	0.06
Serum albumin	0.41 (0.28–0.56)	< 0.001	0.49 (0.35–0.68)	< 0.001
Elevated LDH (> ULN)	2.10 (1.10–4.00)	0.02	2.12 (1.42–4.81)	0.002
Ann-Arbor stage III-IV	2.81 (1.53–5.15)	< 0.001	3.2 (1.83–5.6)	< 0.001
Extranodal lesions	2.24 (1.26–3.97)	0.006	2.49 (1.48–4.19)	< 0.001
Bulky lesion	1.009 (0.54–1.88)	0.98	0.96 (0.55–1.68)	0.89
IPI score	1.50 (1.25–1.79)	< 0.001	1.53 (1.30–1.80)	< 0.001
Diagnosed in other facilities	1.15 (0.61–2.14)	0.67	1.007 (0.57–1.79)	0.98
CHOP (vs dose-reduced)	0.55 (0.34–0.89)	0.02	0.80 (0.53–1.22)	0.31
Nonuse of rituximab	3.76 (1.86–7.58)	< 0.001	2.95 (1.36–6.41)	0.006
Short DTI (0–22 days)	2.89 (1.73–4.83)	< 0.001	2.64 (1.68–4.15)	< 0.001

*PS* performance status, *LDH* lactate dehydrogenase, *ULN* upper limit of normal, *IPI* International Prognostic Index, *DTI* diagnosis-to-treatment interval.

Short DTI (0–22 days), B symptoms, lower serum albumin, higher IPI score, nonuse of rituximab, and dose-reduced THP-COP regimen were evaluated with multivariate analysis for OS. Short DTI (0–22 days) (HR 2.03, 95% CI [1.18, 3.52], p = 0.011), nonuse of rituximab (HR 2.88, 95% CI [1.39, 5.96], p = 0.005), and IPI score (HR 1.25, 95% CI [1.001, 1.55], p = 0.049) were revealed as independent factors affecting OS (Table 3). Short DTI (0–22 days), B symptoms, lower serum albumin, higher IPI score, and nonuse of rituximab were evaluated with multivariate analysis for PFS. Similar as for OS, short DTI (0–22 days) (HR 2.08, 95% CI [1.31, 3.33], p < 0.001), nonuse of rituximab (HR 3.68, 95% CI [1.67, 8.09], p = 0.001), and IPI score (HR 1.45, 95% CI [1.22, 1.72], p = 0.002) were revealed as independent factors affecting PFS (Table 3). With a multivariate analysis including the additional factors such as age, Ann Arbor stage, extranodal lesions, elevated LDH, and poorer PS, the significance of short DTI (0–22 days) and nonuse of rituximab for OS and PFS remained.

**Table 3:** Factors associated with survival by multivariate analysis.

Factor	Hazard ratio (95% CI)	*p*
**Overall survival**		
Short DTI (0–22 days)	2.03 (1.18–3.52)	0.011
Nonuse of rituximab	2.88 (1.39–5.96)	0.005
IPI score	1.25 (1.001–1.55)	0.049
**Progression free survival**		
Short DTI (0–22 days)	2.08 (1.31–3.33)	< 0.001
Nonuse of rituximab	3.68 (1.67–8.09)	0.001
IPI score	1.45 (1.22–1.72)	0.002

*DTI* diagnosis-to-treatment interval, *IPI* International Prognostic Index.

## Discussion

In the present study, we retrospectively evaluated the influence of DTI and other parameters on clinical outcomes in newly diagnosed 199 patients with DLBCL as a single-center analysis. Patients with short DTI (0–22 days) showed significantly worse outcomes (OS, PFS) compared to those with long DTI (over 22 days). However, the association of DTI and outcomes does not seem to be linear. The 2-year OS was lowest for patients with DTI of 5–15 days, highest for those with DTI of 30–40 days, and decreased afterward. The reason of the delay for six patients whose DTI was over 100 days seems to due to comorbidities (n = 5) and poor economy (n = 1). In Japan, all citizens are provided a universal health insurance system and free to choose a medical institution. Although, the economic barriers to medical care are low, it is possible that socioeconomic status and comorbidities are related to too long DTI. Additionally, the nonlinear association of DTI and outcomes is consistent with our feelings as a clinician, because it is easily predicted that DLBCL progresses without treatment and results in worse prognosis.

Although the threshold of acceptable treatment delay is difficult to determine, it seems to be around 30–40 days according to our data.

Hay et al.^8^ analyzed 689 patients with DLBCL using the British Colombia Cancer Registry and reported that DTI longer than 8 weeks is significantly associated with poor OS compared with shorter DTI in patients with DLBCL. They also revealed the association with shorter DTI (0–4 weeks) and poor OS. Patients with intermediate DTI (5–8 weeks) tended to have better OS than those with shorter or longer DTI. Olszewski et al.^9^ reported similar results using data of 104,405 patients with DLBCL from National Cancer Data Base and revealed that a hazard ratio of OS was 1.38 (95% confidence interval 1.28–1.48) in patients with DLBCL who were treated within 7 days compared to those treated > 30 days from diagnosis. The hazard ratio was lowest for patients treated around day 45–60 from diagnosis and increased afterward. Phipps et al.^13^ reported partially consistent results. They analyzed the association with DTI and outcomes in 581 patients with DLBCL who were treated in two major hospitals in Singapore. The median DTI was 14 days. Analyzing DTI as two categories, longer DTI was associated with poor OS and PFS. They did not find an association between short DTI and poor OS. We speculate that they could reveal nonlinear association between DTI and OS, if they analyzed DTI as much more categories.

Three studies reported the association between short DTI and poor outcomes^10–12^. Maurer et al.^10^ analyzed two cohorts, 986 patients in the United States and 1444 patients in Europe. The median DTI was 15 days in the American cohort and 23 days in European cohort. They revealed an association between short DTI and poor event-free survival at 24 months (EFS24). The association remained significant after adjustment for IPI. Examination of functional form revealed approximately linear association of increasing DTI and achieving EFS24. They also analyzed EFS by DTI grouped by week, and DTI over 35 days was shown to be associated with best EFS. Camus et al.^11^ analyzed 345 patients with DLBCL as a real-life monocentric study. The median DTI was 30 days. They revealed an association between shorter DTI and poor OS and PFS. The influence of DTI on OS appears to be a continuous effect. Blunt et al.^12^ analyzed 9446 patients with DLBCL using population-based databases in Canada. The median DTI was 37 days. They revealed an association between shorter DTI and poor OS.

Nikonova et al.^14^ reported an opposite conclusion. They analyzed the impact of treatment delay (the same as DTI) on clinical outcomes in 278 patients with DLBCL. The median DTI was 3 weeks. DTI was divided into three groups (under 1 week, 1–4 weeks, and over 4 weeks) and was analyzed. In conclusion, the delay did not impact OS and PFS.

The discrepancies of results between our study and previous studies may be caused by variance of DTI and statistical method. Contrary to the results of Maurer et al.^10^, our study revealed a nonlinear association between DTI and OS. Although the median DTI of our study is comparable with that of the European cohort in Maurer’s study, ours included more patients with longer DTI. This may have contributed to the decreased OS in patients with longer DTI. Contrary to the results of Nikonova et al^14^, which reported no association between DTI and outcomes, our study and other previous studies^8–12^ revealed the association between short DTI and poor outcomes. This might be due to the difference in categorization of DTI. If they analyzed DTI as much more categories or examine nonlinearity, the result may have been different.

In this study, short DTI was strongly correlated with several known adverse factors. Although the correlation may affect the association between short DTI and poor OS, short DTI remained to be an independent prognostic factor by multivariate analysis. We speculate that patients with short DTI were judged to need urgent treatment due to disease aggressiveness which is not appropriately included in standard prognostic tools. Therefore, in addition to well-known prognostic factors, DTI should be considered when reporting outcomes of clinical trials.

More challenging, patients who require treatment soon after diagnosis cannot participate in prospective clinical trials which usually take time to perform additional examination or randomization. Patients with short DTI tend to have poor prognostic factors, and there are high unmet medical needs. In clinical trials to examine new treatment strategies and solve such unmet medical needs, enrollment of patients with short DTI is essential. Researchers should consider DTI as one of the important prognostic factors and plan clinical trials to be able to enroll patients who require treatment soon after diagnosis.

This study has several limitations due to its retrospective nature. First, it is possible that unmeasured residual confounding factors may affect outcomes. There are no data about DLBCL subtypes by gene expression profiles or immunophenotyping. In addition, multivariate analysis is not able to fully compensate for differences between patients due to unexpected covariates. Second, we could not collect data about causes of treatment delay, because it was not fully recorded. It should be noted that there is a possible association between the cause of treatment delay and outcomes. However, performing prospective randomized study is not possible due to ethical problem. Nonetheless, this study makes an important contribution in that it revealed the importance of DTI.

In conclusion, our study confirmed the importance of DTI. Short DTI (0–22 days) is associated with poor OS and PFS. Besides that, it is possible that too long DTI is a poor prognostic factor. DTI should be considered when reporting outcomes of clinical trials.

## Data Availability

The datasets generated during and analysed during the current study are available from the corresponding author on reasonable request.
